# Supplementary material for: Population genomics and antimicrobial resistance in Corynebacterium diphtheriae
Source: Genome Med. 2020 Nov 27;12:107. doi: 10.1186/s13073-020-00805-7 (PMC7694903; doi:10.1186/s13073-020-00805-7)
Supplement: Supplementary file 4 — Additional file 4. Contains Supplementary Figures S1 to S14. [file 13073_2020_805_MOESM4_ESM.pdf]

SUPPLEMENTARY FIGURES

Population genomics and antimicrobial resistance in *Corynebacterium diphtheriae*

Melanie Hennart[1,2], Leonardo G. Panunzi[1,3], Carla Rodrigues[1], Quentin Gaday[4], Sarah L. Baines[5], Marina Barros-Pinkelnig[1], Annick Carmi-Leroy[1,6], Melody Dazas[1,6], Anne-Marie Wehenkel[4], Xavier Didelot[7], Julie Toubiana[1,6,8], Edgar Badell[1,6] and Sylvain Brisse[1,6,*]

[1] Institut Pasteur, Biodiversity and Epidemiology of Bacterial Pathogens, Paris, France.

[2] Sorbonne Université, Collège doctoral, F-75005 Paris, France

[3] Institut Français de Bioinformatique, CNRS UMS 3601, Evry, France

[4] Unité de Microbiologie Structurale, Institut Pasteur, CNRS UMR 3528, Université de Paris, F-75015 Paris, France.

[5] Doherty Applied Microbial Genomics, Department of Microbiology & Immunology, The University of Melbourne at The Peter Doherty Institute for Infection & Immunity, Melbourne, Victoria, Australia

[6] Institut Pasteur, National Reference Center for Corynebacteria of the diphtheriae complex, Paris, France.

[7] School of Life Sciences and Department of Statistics, University of Warwick, United Kingdom

[8] Université de Paris, Department of General Pediatrics and Pediatric infectious diseases, Hôpital Necker-Enfants malades, APHP, Paris France

*Corresponding author. Sylvain Brisse. Institut Pasteur, Biodiversity and Epidemiology of Bacterial Pathogens, Paris, France. E-mail: sbrisse@pasteur.fr; Tel: +33 1 45 68 83 34

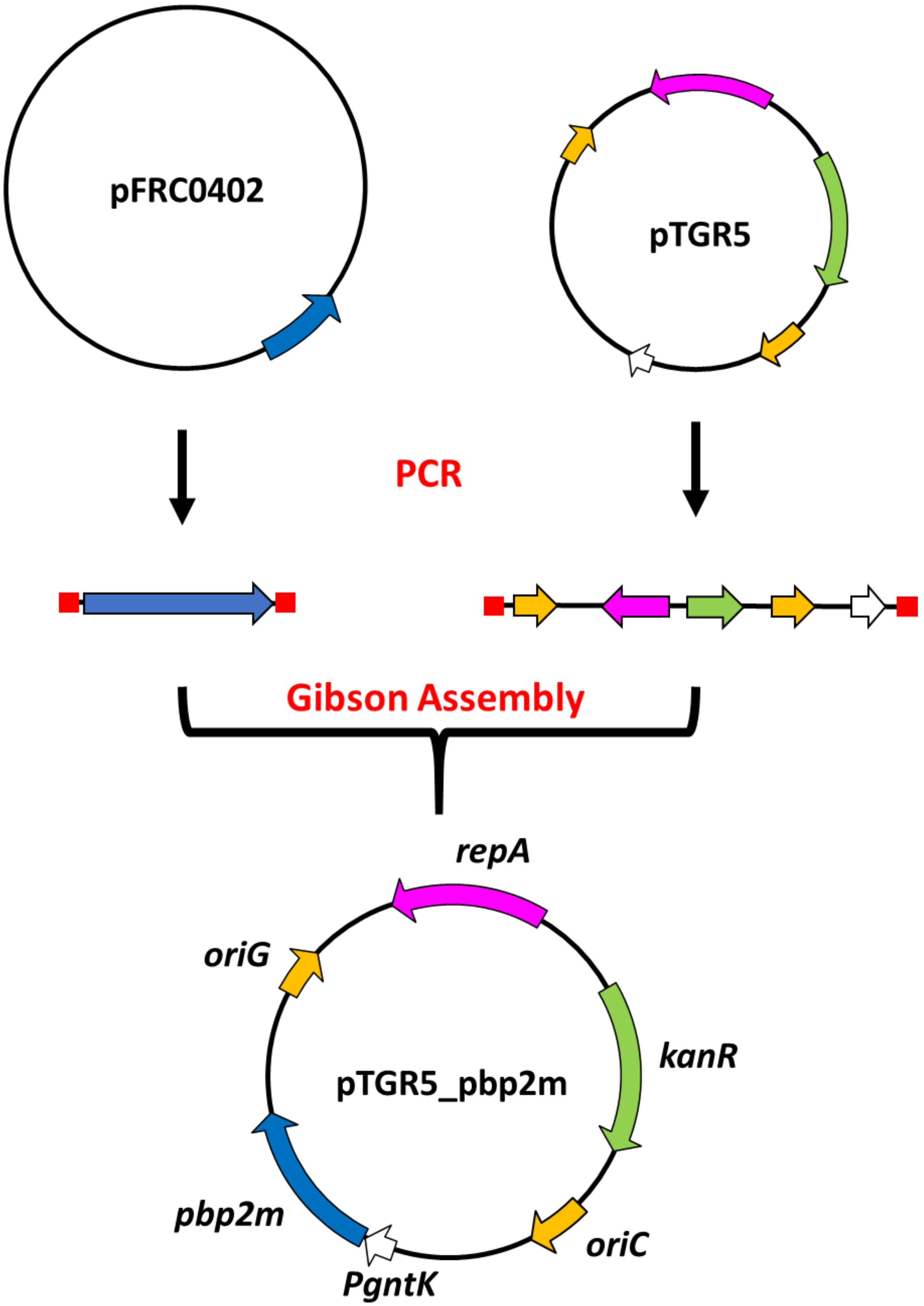

Fig S1. Construction strategy of plasmid pTGR5_pbp2m

The *pbp2m* gene was PCR amplified and combined with plasmid pTGR5 using Gibson assembly as indicated.

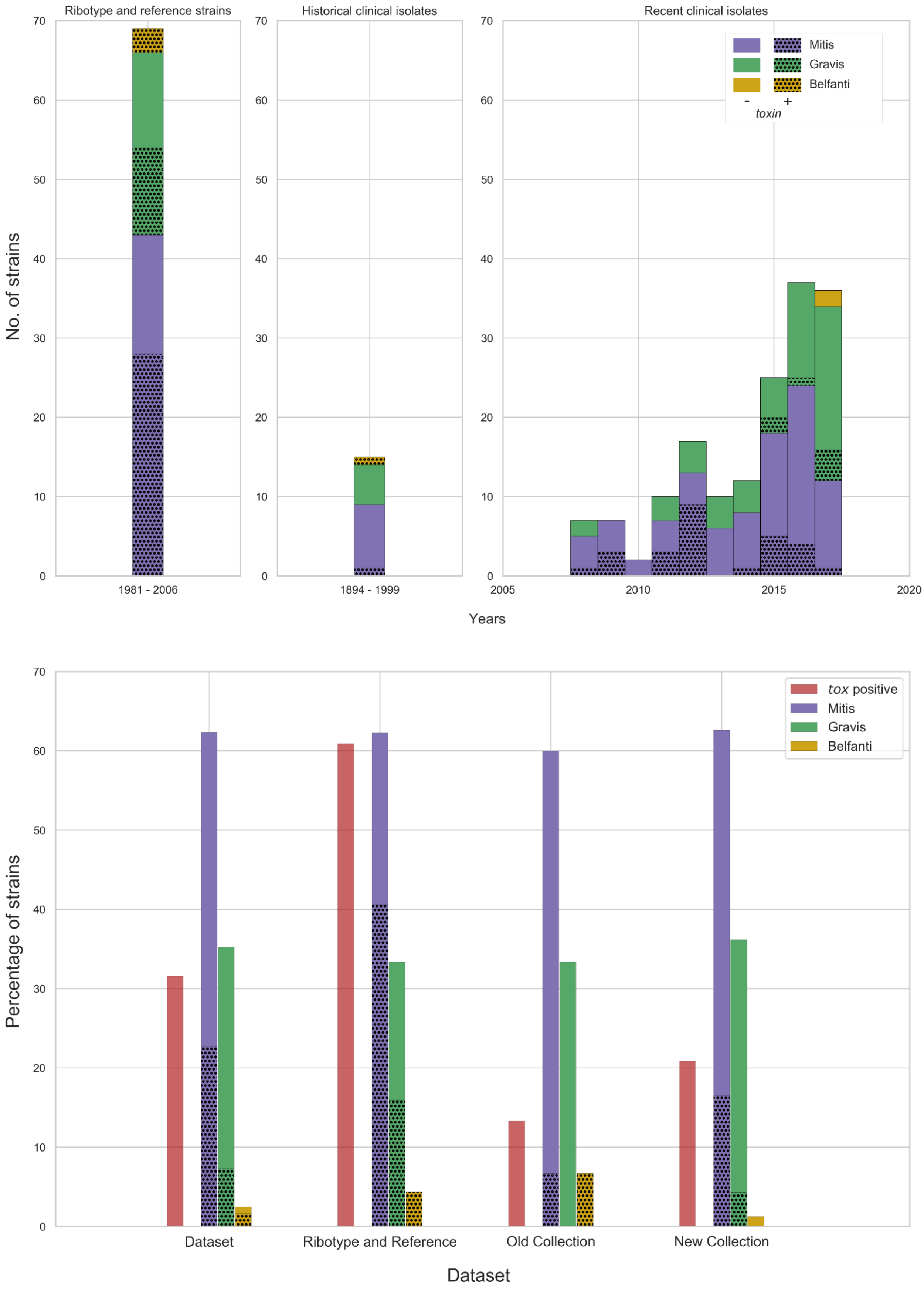

Fig S2. Biovar and *tox* status of the three strain subsets

In the upper panel, the numbers of strains are given separately for the three subsets of strains; the recent clinical isolates one (right hand side) is broken down by individual year. Colors correspond to biovars (see key) and shaded areas denote *tox*-positive isolates. In the lower panel, the percentage of *tox*-positive strains (red bars) and of *tox*-positive or *tox*-negative strains per biovar (see key), are given for the entire dataset and for the three subsets separately; shaded sectors correspond to *tox*-positive strains within each biovar.

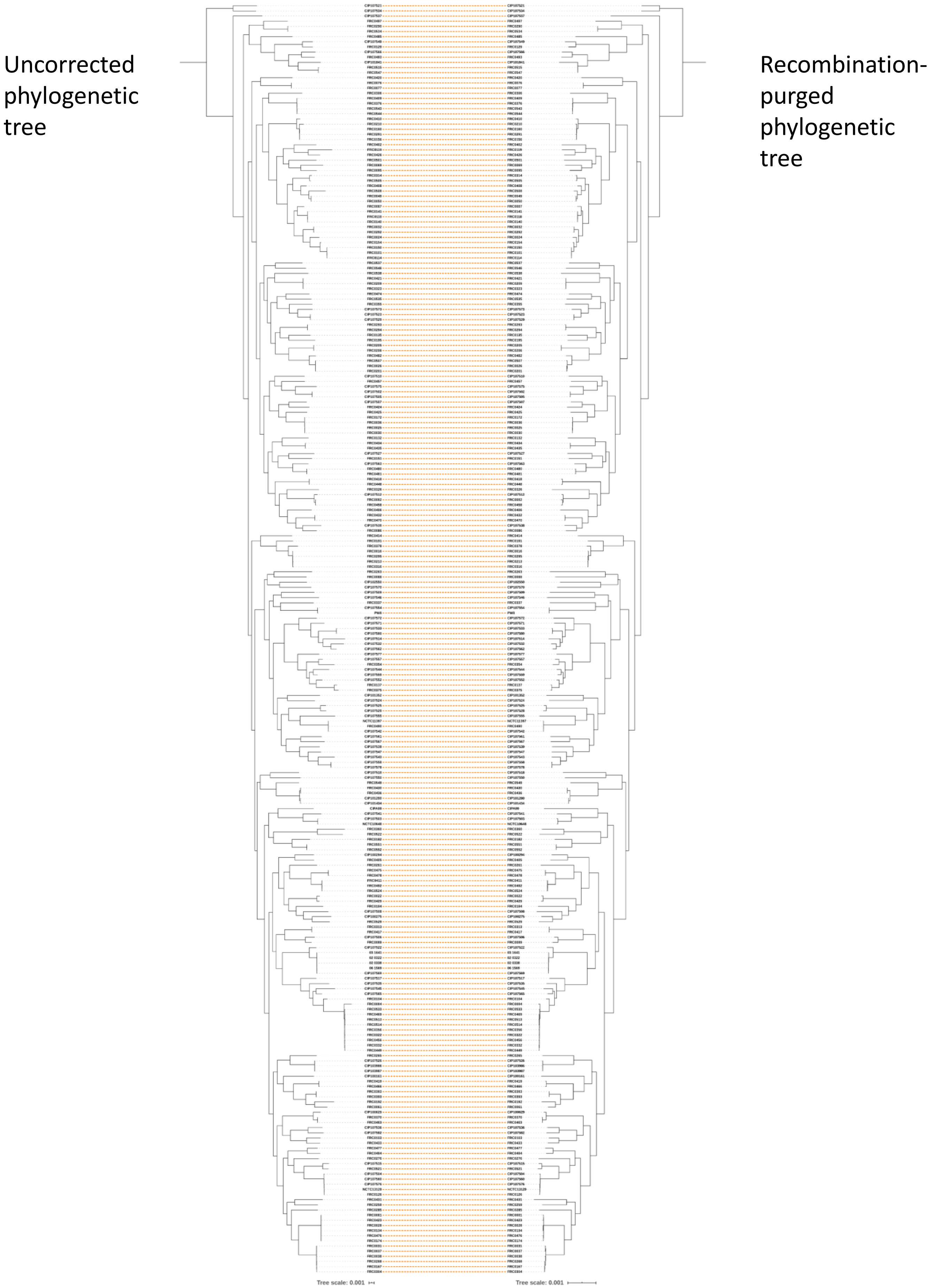

Fig S3. Tanglegram

Tanglegram comparing the uncorrected phylogenetic tree (obtained with PhyML on the core genome alignment; left) and the recombination-purged tree obtained using ClonalFrameML (right). The topologies were identical, but the branch lengths were shorter after purging recombined regions (note that the scale of both trees is different).

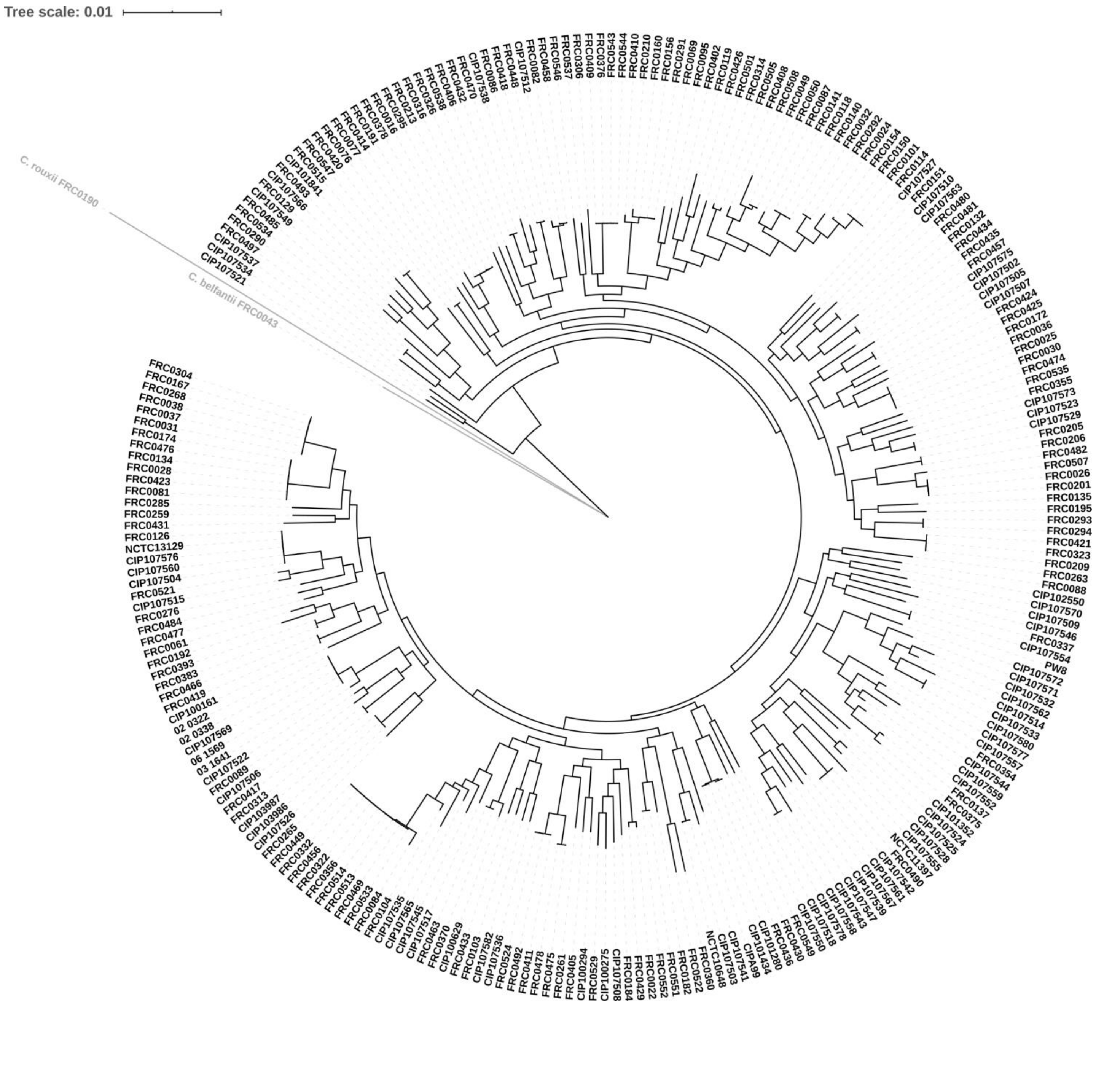

Fig S4. Phylogeny with the type strains of *C. belfantii* and *C. rouxii*.

The phylogeny was obtained using PhyML from the core genome alignment obtained using ROARY. This tree indicated the root of the *C. diphtheriae* tree to be located on the branch leading to the two early-diverging strains CIP107521 and CIP107534.

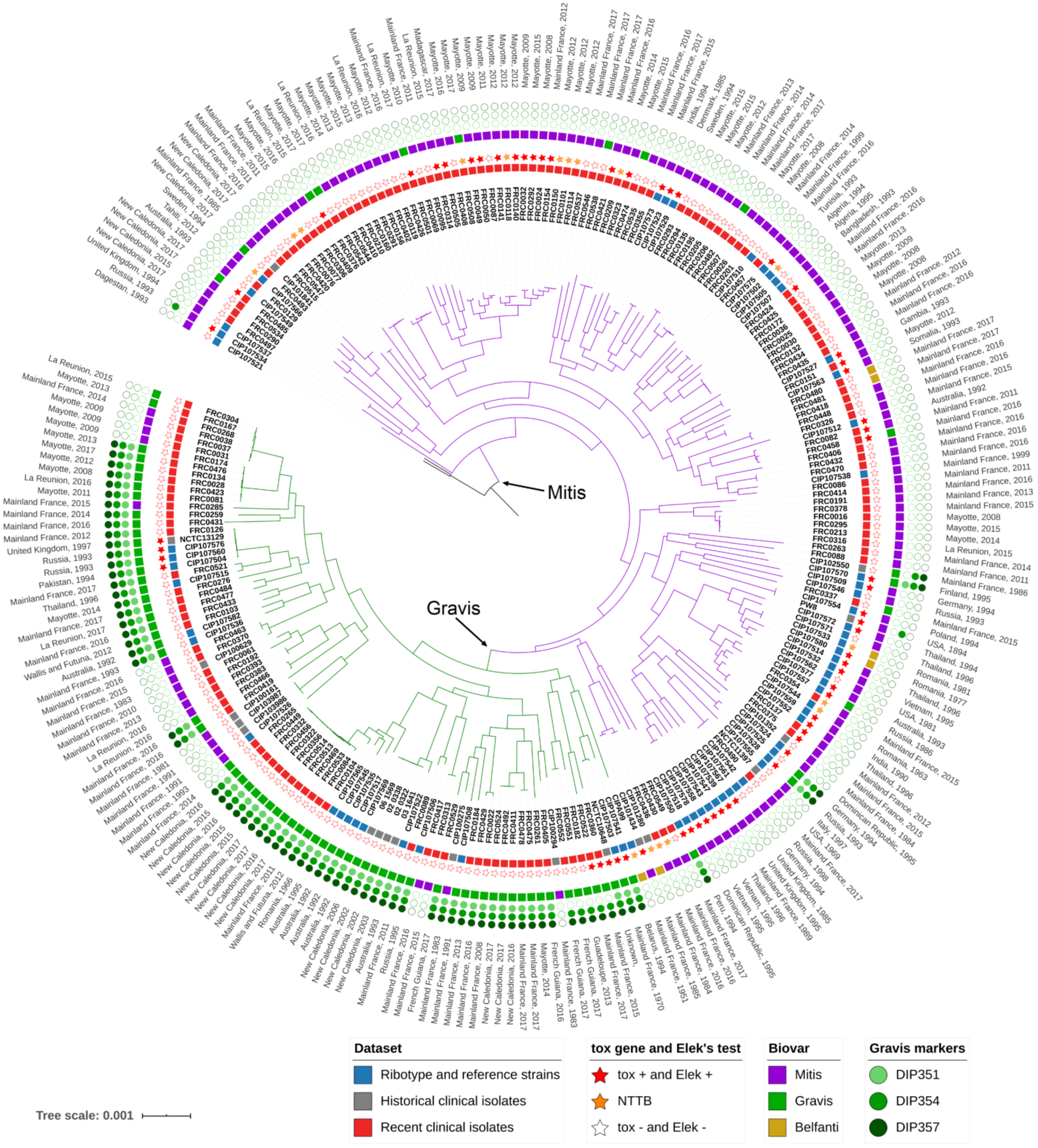

Fig S5. Phylogenetic tree of *C. diphtheriae,* with isolates names
The phylogenetic tree and outer information correspond to those in Figure 2, with the addition of isolates names, geographic origins and year of isolation.

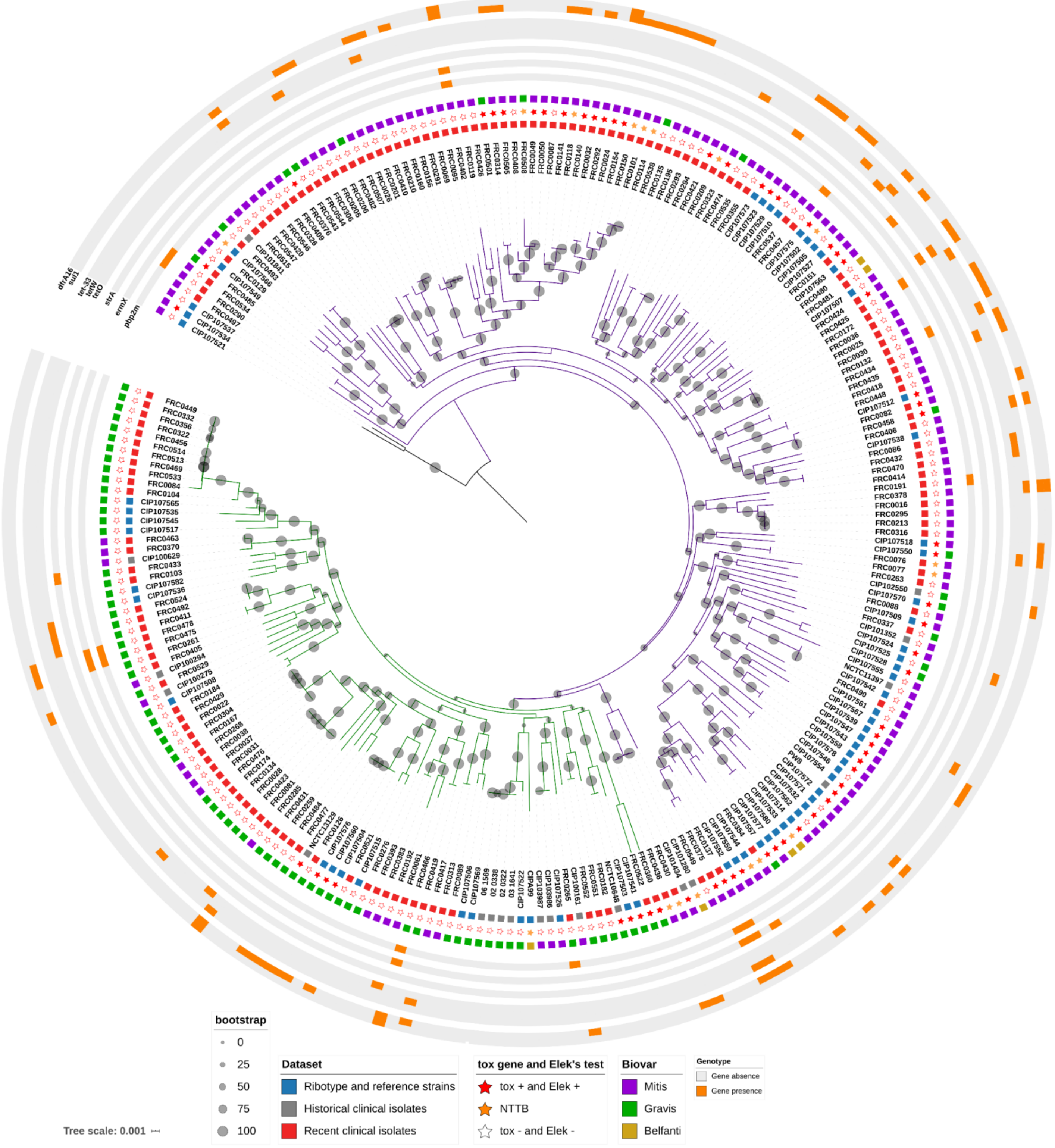

Fig S6. Phylogeny with branch support.

The phylogenetic tree was obtained using IQtree version 2 from the core genome alignment produced using ROARY. Bootstrap values were obtained using IQtree bootstrap resampling option based on 58 replicates. Only bootstrap percentage values between 50 and 100 are represented at the nodes (see key). The circles around the tree display the dataset (internal circle), *tox* gene presence and toxin production (second circle), biovar (third circle) and the presence of 8 selected antimicrobial resistance genes (see names besides each circle).

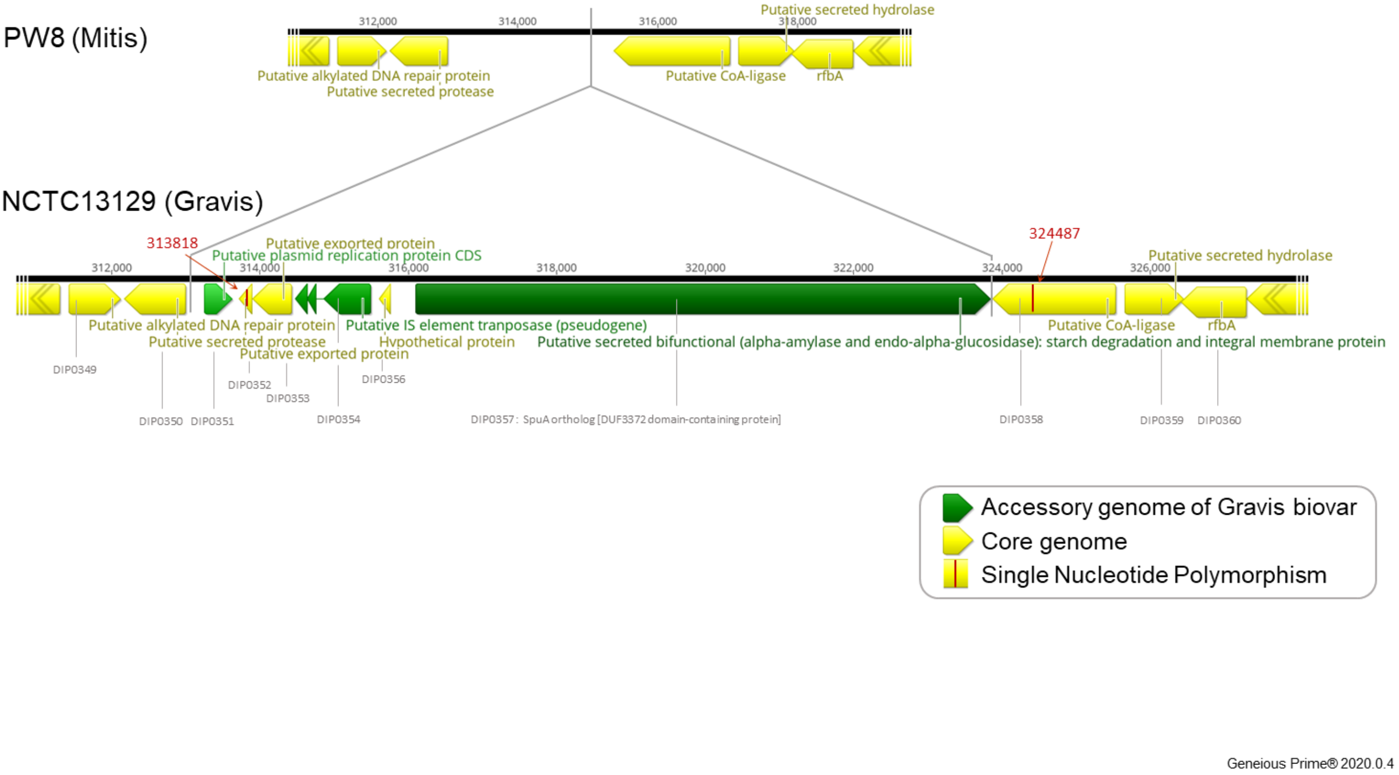

Fig S7. Genomic difference between reference strains PW8 (biovar Mitis) and NCTC13129 (Gravis)

The genomic region of approx. 10 kb inserted in biovar Gravis strain NCTC13129 includes genes DIP0351, DIP0354 and *spuA* (DIP0357); these three accessory genes are strongly associated with biovar Gravis, as is the SNP at position 324,487.

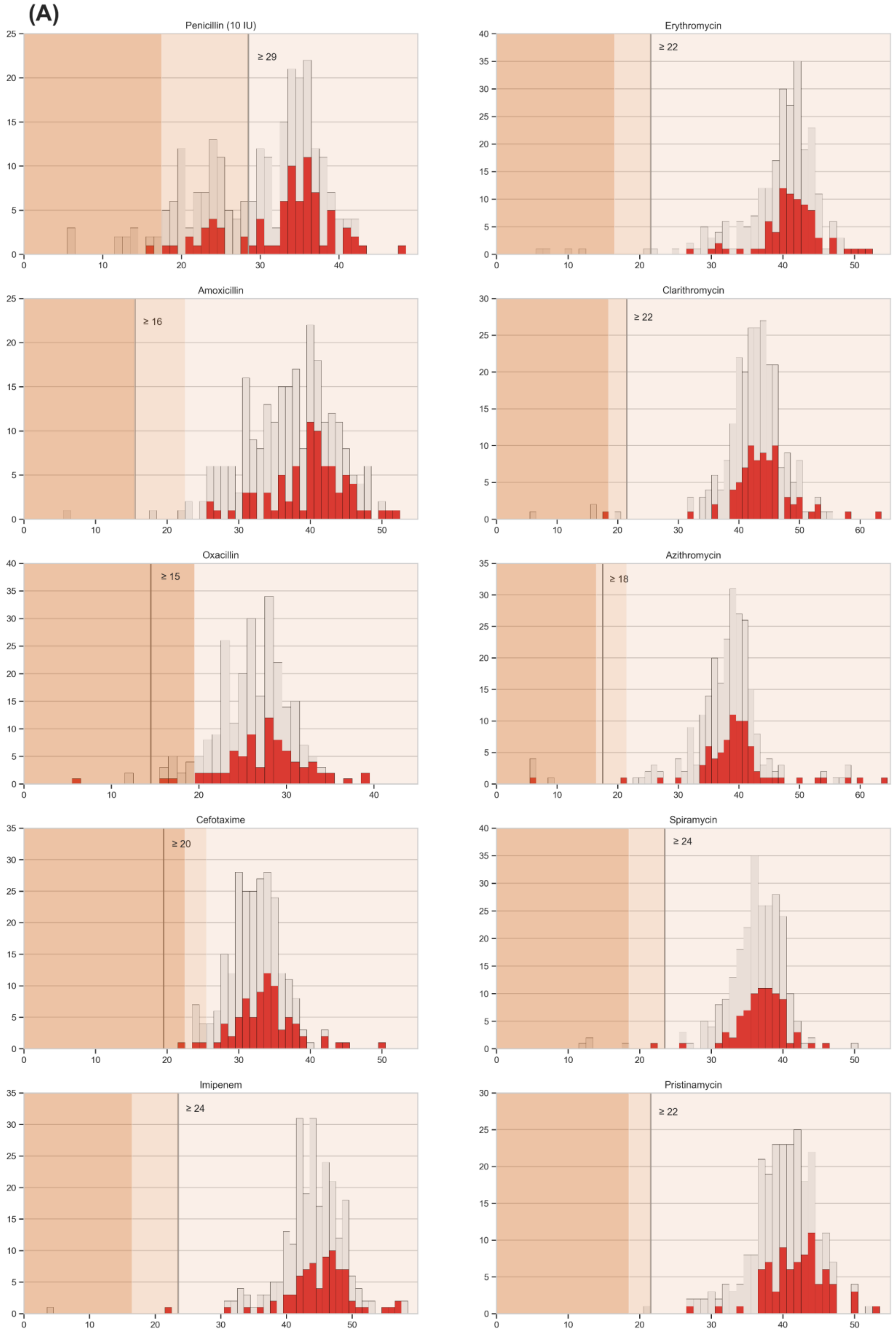

Fig S8. The distributions of zone diameter values for 19 antimicrobial agents, colored by the presence of the *tox* gene

A: beta-lactams, macrolides and pristinamycin. **B**: other agents. X-axis: diameter in mm; Y-axis: number of strains. Colors inside the bars represent *tox*-positive isolates (red) or *tox*-negative isolates (grey). The three background colors represent the categorical interpretations according to EUCAST: resistant (salmon, left), intermediate (lighter salmon, middle) and susceptible (pale beige, right). The grey vertical bar corresponds to the proposed tentative ecological cutoff.

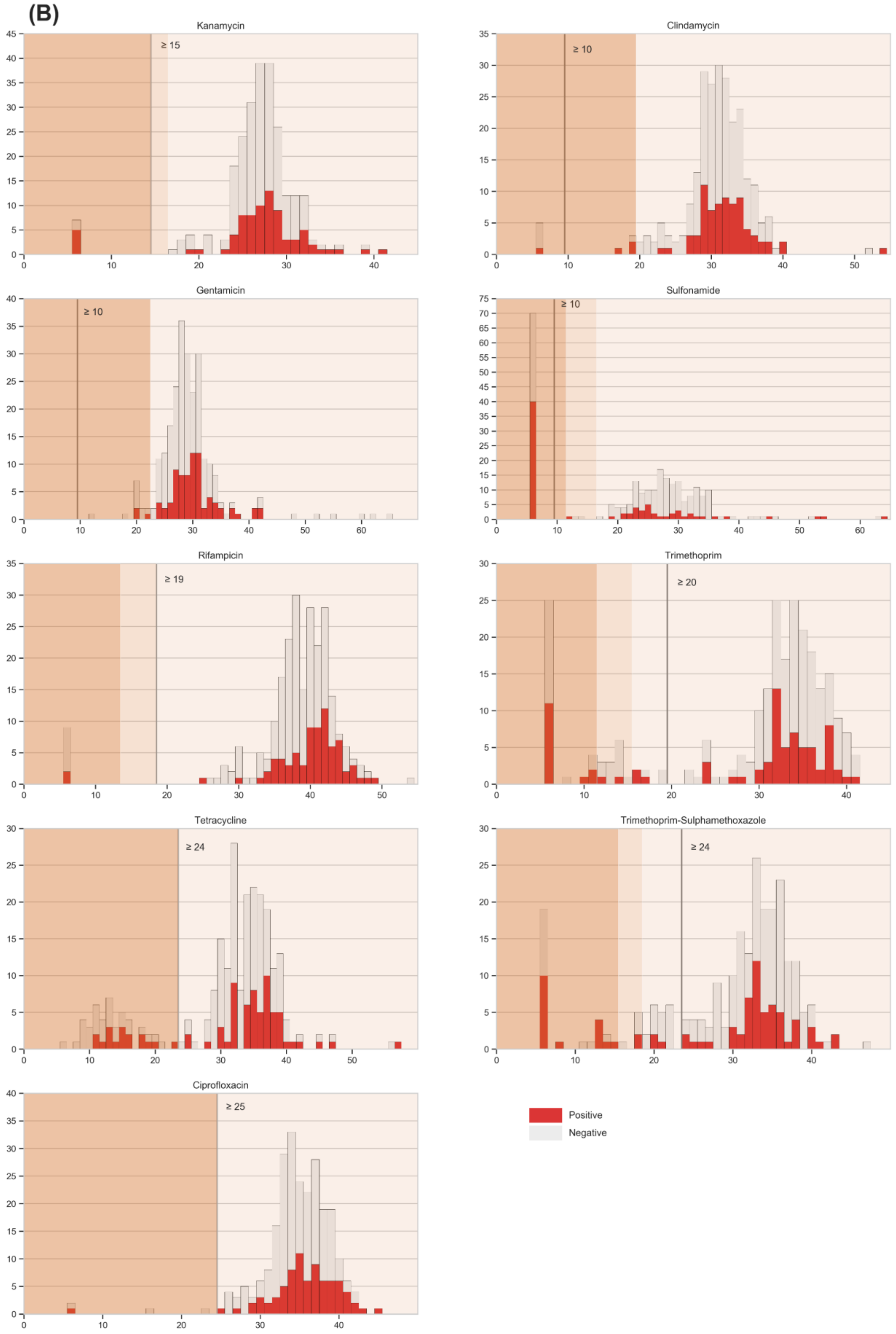

Fig S8. The distributions of zone diameter values for 19 antimicrobial agents, colored by the presence of the *tox* gene

A: beta-lactams, macrolides and pristinamycin. **B**: other agents. X-axis: diameter in mm; Y-axis: number of strains. Colors inside the bars represent *tox*-positive isolates (red) or *tox*-negative isolates (grey). The three background colors represent the categorical interpretations according to EUCAST: resistant (salmon, left), intermediate (lighter salmon, middle) and susceptible (pale beige, right). The grey vertical bar corresponds to the proposed tentative ecological cutoff.

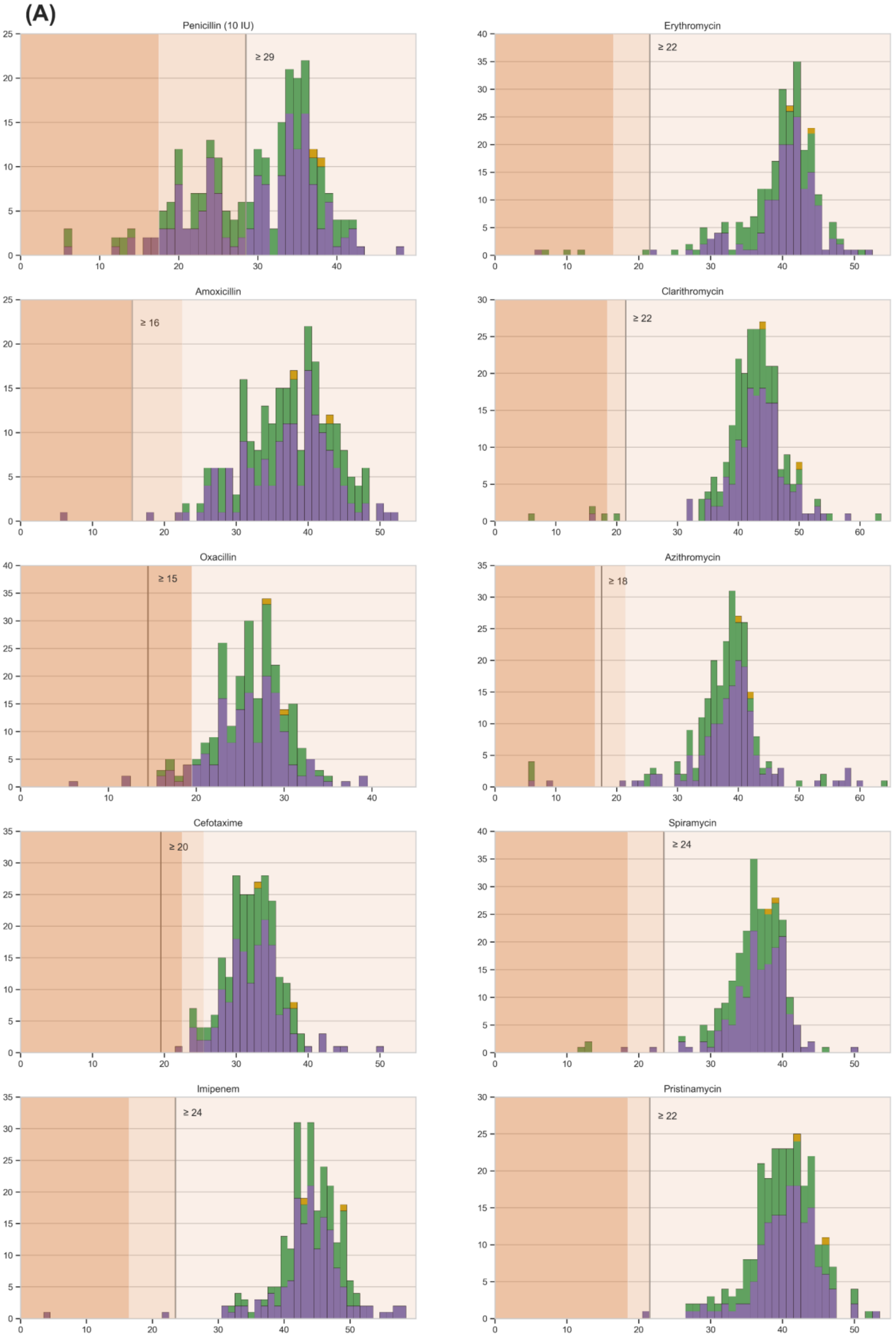

Fig S9. The distributions of zone diameter values for 19 antimicrobial agents, colored by the main lineages (Mitis and Gravis)

A: beta-lactams, macrolides and pristinamycin. **B**: other agents. X-axis: diameter in mm; Y-axis: number of strains. Colors inside the bars represent the two main lineages (see key in panel B). The three background colors represent the categorical interpretations according to EUCAST: resistant (salmon, left), intermediate (lighter salmon, middle) and susceptible (pale beige, right). The grey vertical bar corresponds to the proposed tentative ecological cutoff.

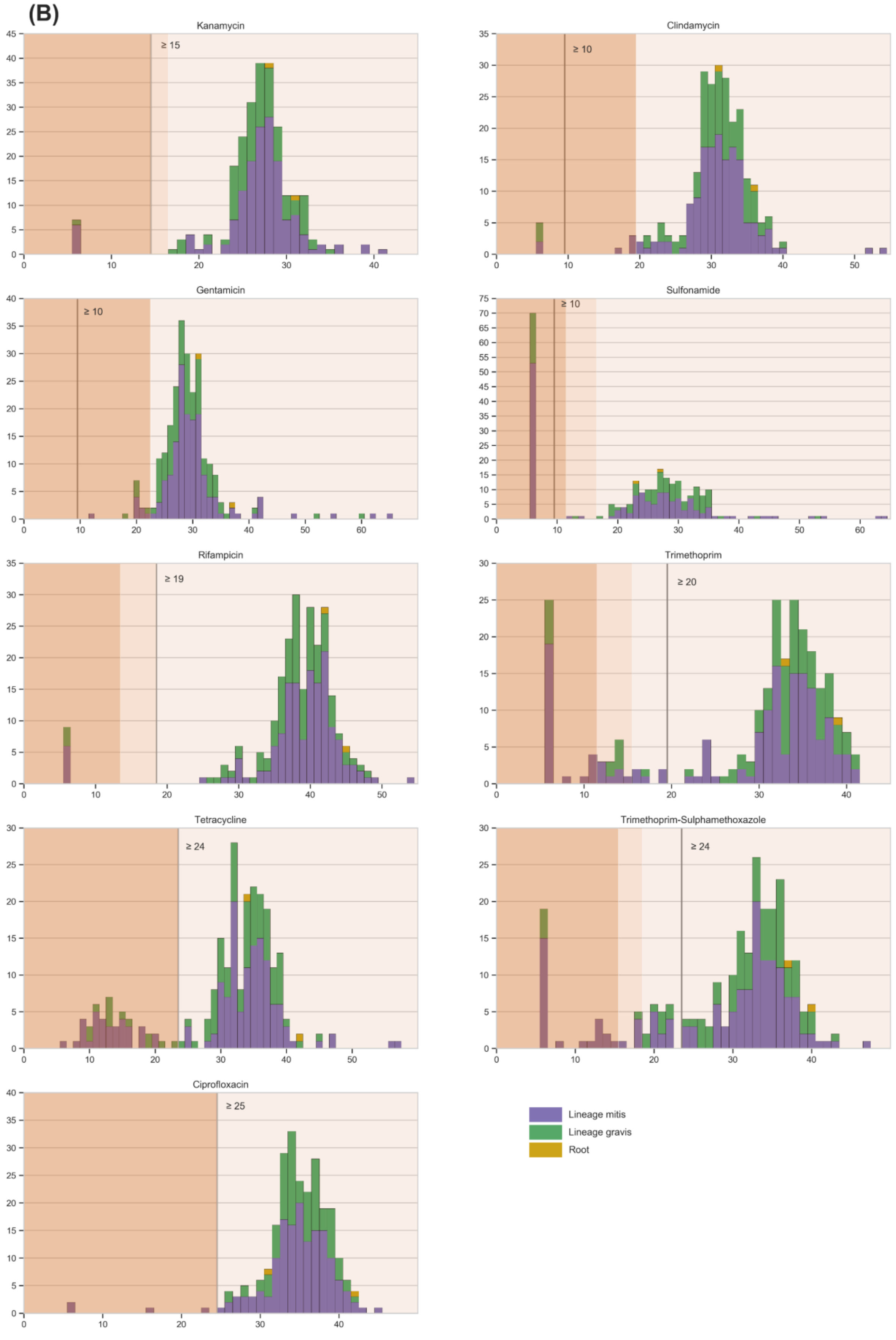

Fig S9. The distributions of zone diameter values for 19 antimicrobial agents, colored by the main lineages (Mitis and Gravis)

A: beta-lactams, macrolides and pristinamycin. **B**: other agents. X-axis: diameter in mm; Y-axis: number of strains. Colors inside the bars represent the two main lineages (see key in panel B). The three background colors represent the categorical interpretations according to EUCAST: resistant (salmon, left), intermediate (lighter salmon, middle) and susceptible (pale beige, right). The grey vertical bar corresponds to the proposed tentative ecological cutoff.

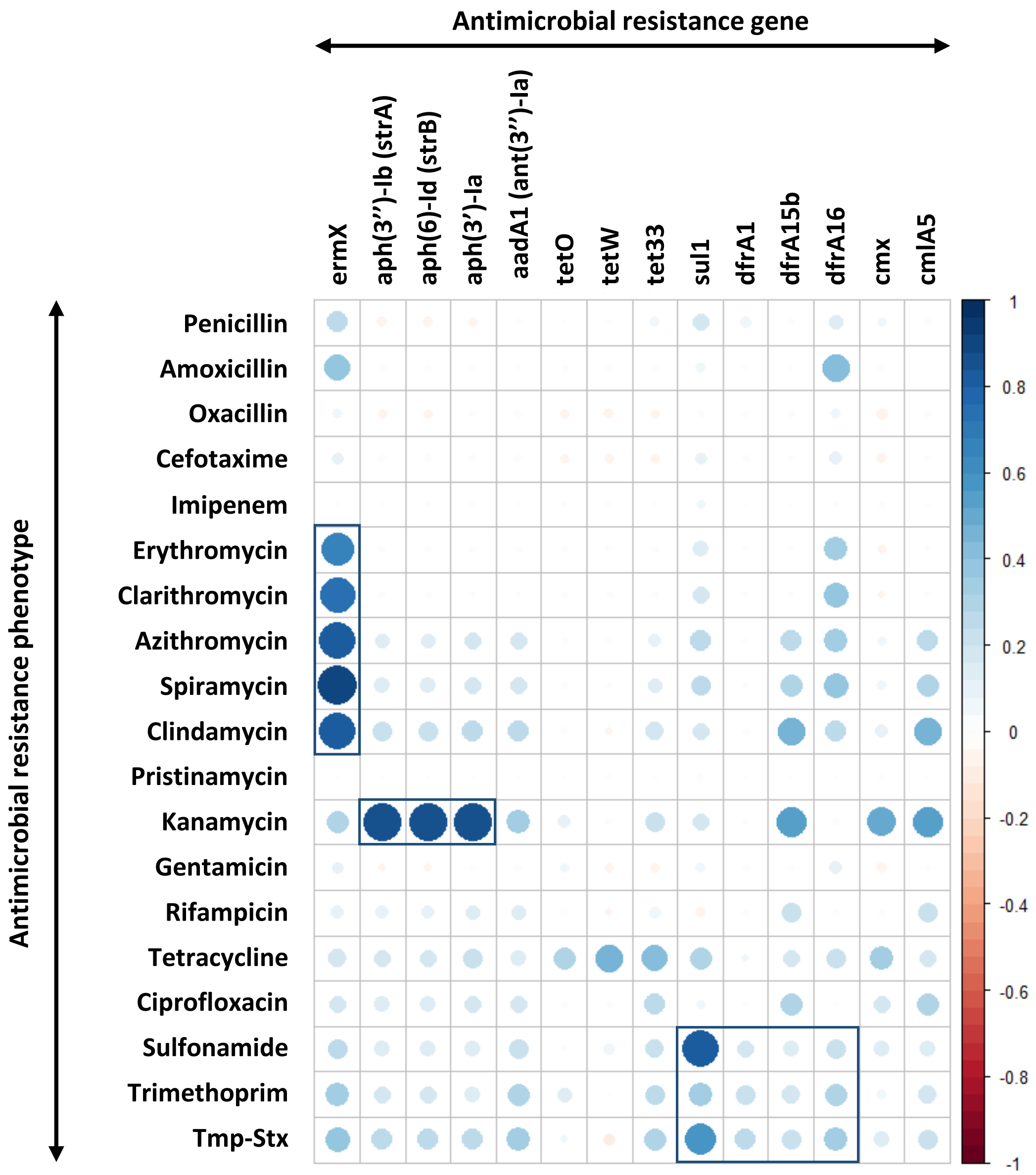

Fig S10. Correlation plot of antimicrobial resistance phenotypes and genotypes. The correlation matrix between antimicrobial resistance genotype and phenotype is based on the correlation for binary variables (in the case of resistance genes: 1, presence; 0, absence; in the case of antimicrobial drugs: 1, resistant/intermediate; 0, susceptible) using the 'corr.test' function (Pearson method, which for a pair of binary variables equates to the Phi coefficient) from the 'corrplot' R package. Significant correlations were visualized utilizing the 'corrplot' function from the same package. Blank squares represent correlations without statistical significance ($p > 0.05$). Positive correlation is depicted by blue circles, whereas red circles represent significant negative correlation. The size and strength of color represent the numerical value of the Phi correlation coefficient. Black rectangles group genes commonly found together in the same strain. Genes *cmx* and *cmlA5* are known to be associated with chloramphenicol resistance, which was not tested here.

Fluoroquinolone

Ciprofloxacin

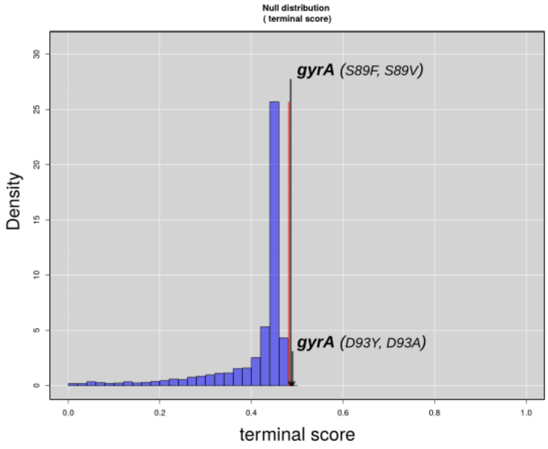

RNA metabolism

Rifampicin

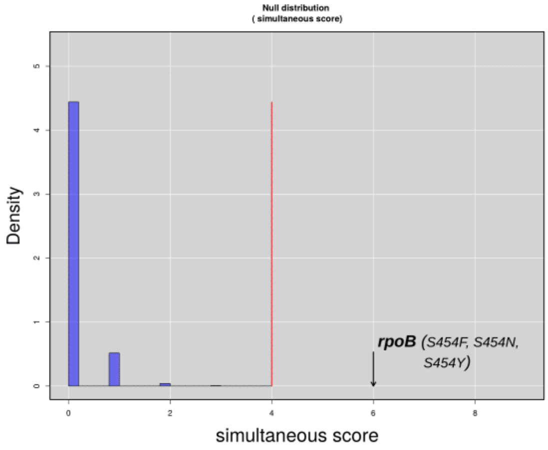

Folic acid metabolism

Trimethropin

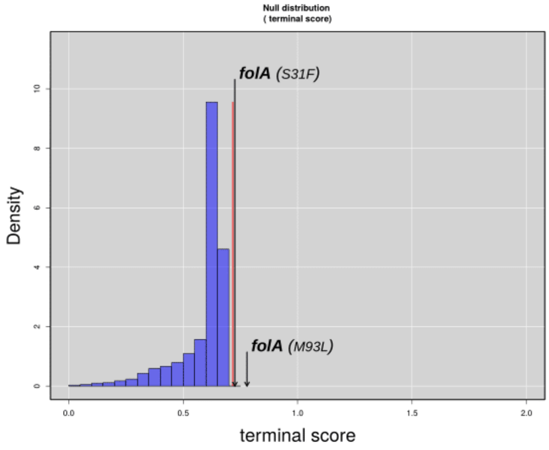

Fig S11. treeWAS results plots for ciprofloxacin, rifampicin and trimethoprim
Distribution of treeWAS scores obtained for genome-wide SNPs in association with ciprofloxacin, rifampicin and trimethoprim. Significant SNPs in *gyrA*, *rpoB* and *folA* are indicated.

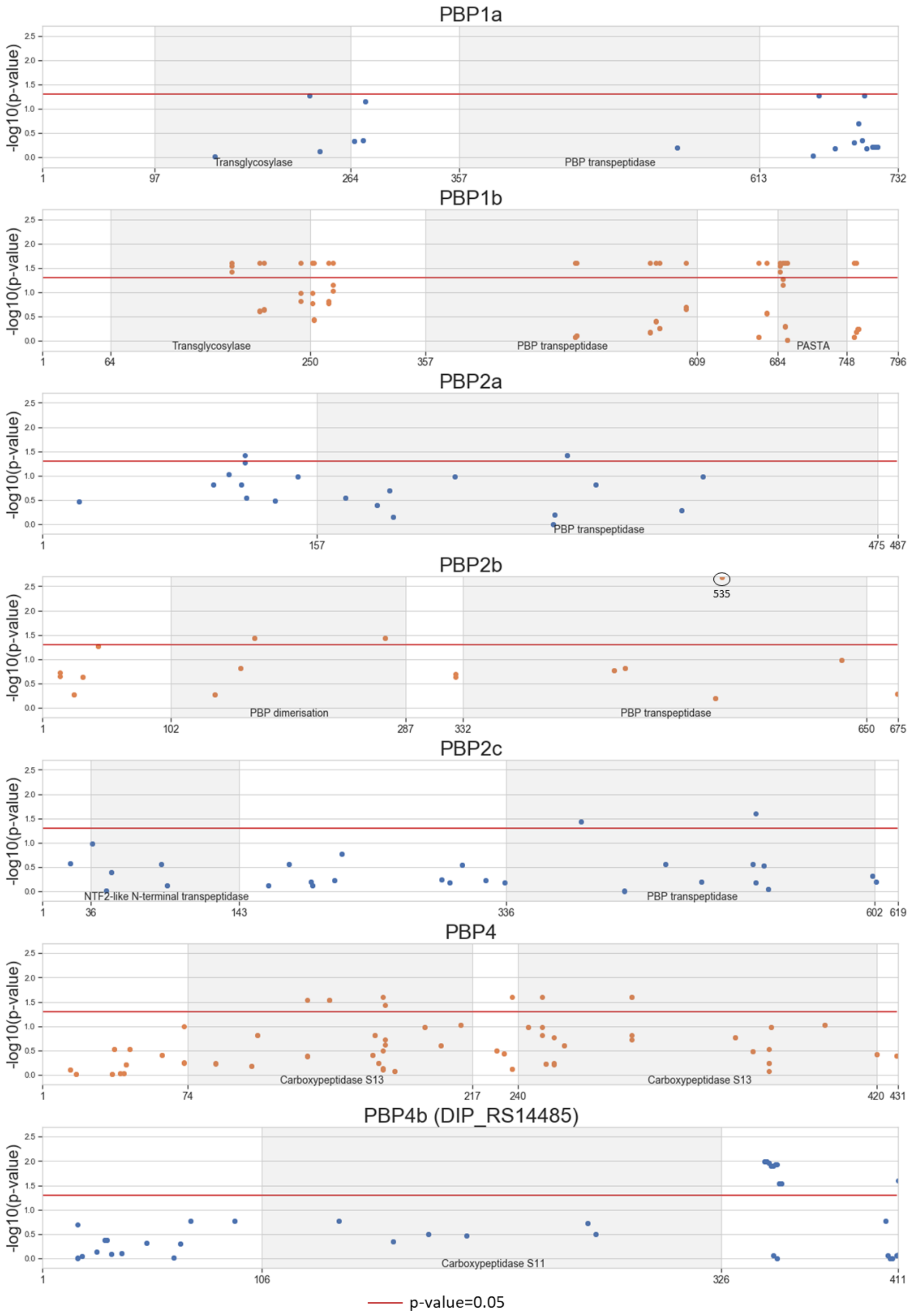

Fig S12. treeWAS results for amino acid polymorphisms in the chromosomal PBP coding genes of *C. diphtheriae*

Statistical significance of the treeWAS subsequent score obtained when testing the association of deduced amino-acid alterations in the seven chromosomal PBP sequences, and penicillin resistance phenotype. Within each of the seven panel the X-axis represent the amino acid sequence (numbers: AA positions), and the Y-axis the –log10(p-value). The positions of transglycosylase, transpeptidase, carboxypeptidase or other relevant domains of the PBP are shaded in grey. SNPs are represented as blue or orange circles (in alternance) at their corresponding position. The red bar indicates the 0.05 p-value position. The most significant SNP, at position 535 of PBP2b, is circled.

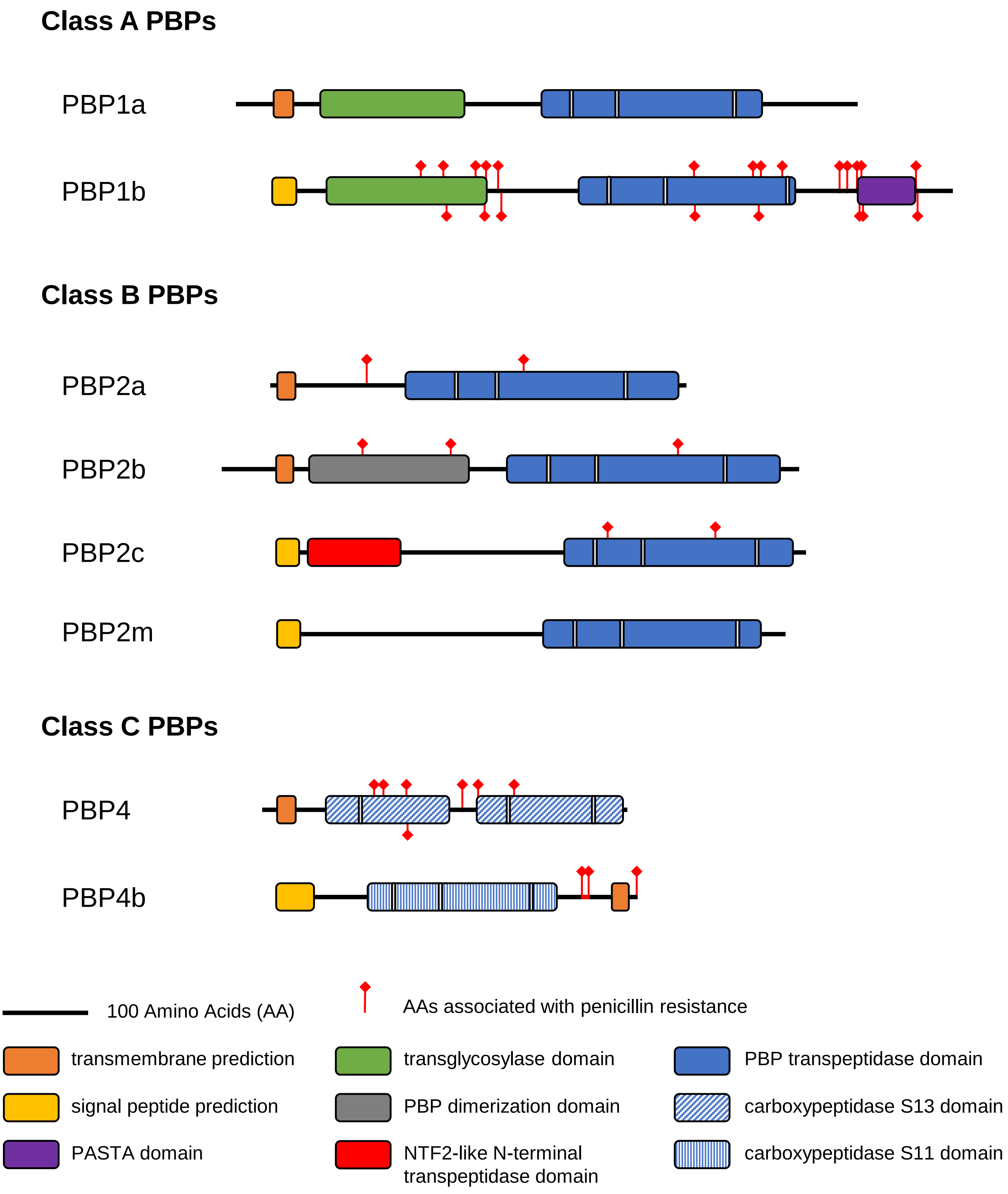

Fig S13. Functional annotation and mapping of significant SNPs associated with *C. diphtheriae* chromosomal PBPs.
Conserved transpeptidation motifs SxxK SxN KTG are indicated on the transpeptidase and carboxypeptidase domains by white lines. Significant SNPs associated with penicillin resistance are indicted by red pins. The PBPs correspond to the following genes: *pbp1a* (DIP2294), *pbp1b* (DIP0298), *pbp2a* (DIP0055), *pbp2b* (DIP1604), *pbp2c* (DIP1497), *pbp4* (DIP2005) and *pbp4b* (RS14485 = DIP0637). Pbp2m was not analyzed for amino-acid changes associated with penicillin resistance, as it corresponds to an accessory PBP.

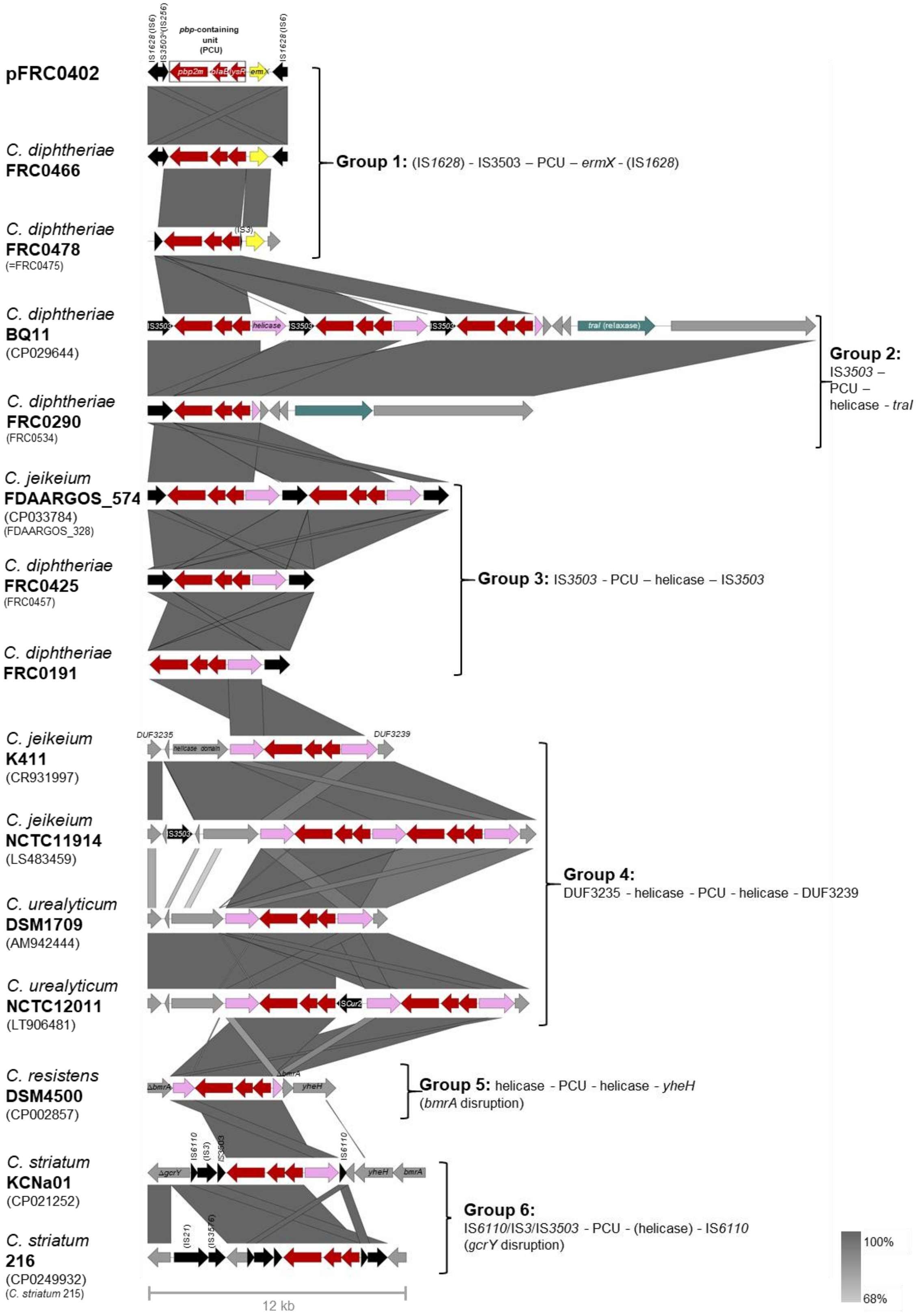

Fig S14. Genetic context of the pbp2m gene in *Corynebacterium*

The genomic context of *pbp2m* in *C. diphtheriae* and other *Corynebacterium* strains that possess this gene is given for representative genomes of the diversity that was found. Genes *pbp2m*, *blaB* and *lysR* are represented with a dark red background; these three genes were always associated and constitute the *pbp*-containing unit (PCU). Gene *ermX* is in yellow. A putative helicase often associated with the PCU is represented in pink; a relaxase gene is shaded in green. Black arrows represent insertion sequence genes. Six groups were defined based on conserved features, as indicated. Dark grey parallelepiped joining different genomes represent homology levels, as indicated in the gradient key. Strains of the present study with identical structures as those represented are indicated in parentheses below the strain name of the representative genome. The scale bar represents 12 kb.
